# Providing visualisation support for the analysis of anatomy ontology data

**DOI:** 10.1186/1471-2105-6-74

**Published:** 2005-03-24

**Authors:** Aba-Sah Dadzie, Albert Burger

**Affiliations:** 1 School of Mathematical and Computer Sciences, Heriot-Watt University, Edinburgh EH14 4AS, Scotland; 2 Medical Research Council, Human Genetics Unit, Western General Hospital, Edinburgh EH4 2XU, Scotland

## Abstract

**Background:**

Improvements in technology have been accompanied by the generation of large amounts of complex data. This same technology must be harnessed effectively if the knowledge stored within the data is to be retrieved. Storing data in ontologies aids its management; ontologies serve as controlled vocabularies that promote data exchange and re-use, improving analysis.

The Edinburgh Mouse Atlas Project stores the developmental stages of the mouse embryo in anatomy ontologies. This project is looking at the use of visual data overviews for intuitive analysis of the ontology data.

**Results:**

A prototype has been developed that visualises the ontologies using directed acyclic graphs in two dimensions, with the ability to study detail in regions of interest in isolation or within the context of the overview. This is followed by the development of a technique that layers individual anatomy ontologies in three-dimensional space, so that relationships across multiple data sets may be mapped using physical links drawn along the third axis.

**Conclusion:**

Usability evaluations of the applications confirmed advantages in visual analysis of complex data. This project will look next at data input from multiple sources, and continue to develop the techniques presented to provide intuitive identification of relationships that span multiple ontologies.

## Background

Improvements in technology have resulted in the ability to perform increasingly complex experimental research, generating large amounts of multi-dimensional data. Research in biology today involves the exploration and analysis of the large, shared databases available to the wider scientific community. This reduces duplication of effort and allows more extensive research to be done, by providing independent data sources for verifying hypotheses formulated.

Conversely, technology has been unable to manage the data it has spawned; data management, analysis and visualisation tools struggle to meet requirements [1]. This is due largely to the constantly evolving information needs of the biologists working with the increasingly large amounts of heterogeneous data generated [2]. Bioinformatics developed to exploit information technology in biological data analysis [3,4].

This paper looks at the development of visual solutions for intuitive analysis of anatomy ontologies. The primary data source is the Edinburgh Mouse Atlas Project (EMAP), developed at Edinburgh's Medical Research Council's (MRC) Human Genetics Unit (HGU), documenting the developmental stages of the mouse embryo.

Prototypes are being developed for visualisation in 2D (two-dimensions) and 3D, to highlight relationships between and within different anatomy components. This will help uncover knowledge about gene expression, structure and function as cells and tissues evolve into defined organs, to track normal development and evolution.

### Visual solutions for effective analysis of biological data

At least a basic understanding of the structure of the anatomy ontology data is required to provide effective design and development with mappings to spatial representations that capture users' mental models of the data. Visualisations generated should provide biologists with data overviews, followed by the ability to study regions of interest (ROIs) in detail, within the context of the overall data set, to highlight patterns within the data [5,6]. Support for interactive data exploration should be provided; functionality for browsing and searching and for manipulation of data structures allows analysis from multiple perspectives.

Visual encoding of textual data exploits humans' highly developed perceptual abilities to decrease the cognitive load associated with complex data analysis [6,7]. A number of visualisation methods and techniques already exist for complex data analysis, both within and outwith the field of bioinformatics, including 2D and 3D scatter plots, self-organising maps (SOMs), parallel coordinates, 2D and 3D hierarchical graphs, information maps, murals and cubes, perspective walls, virtual landscapes, cityscapes, and physical space metaphors such as rooms, windows and desktops. Hyperbolic or fish-eye views and lenses, magic and semantic lenses, and dynamic query systems aid detailed study of regions of interest (ROIs) especially in large data sets. [3,6,8-10]

In order to ascertain what would provide, individually or in concert, optimal visual data analysis solutions for the study of anatomy ontologies, it is necessary to assess existing tools and techniques to determine their applicability to the data sets of interest and the tasks biologists perform. It is important to provide analysis solutions that the different target users with varying research backgrounds are able to use to interpret data required for their work effectively. [11]

### The Edinburgh Mouse Atlas Project

**Figure 1:**
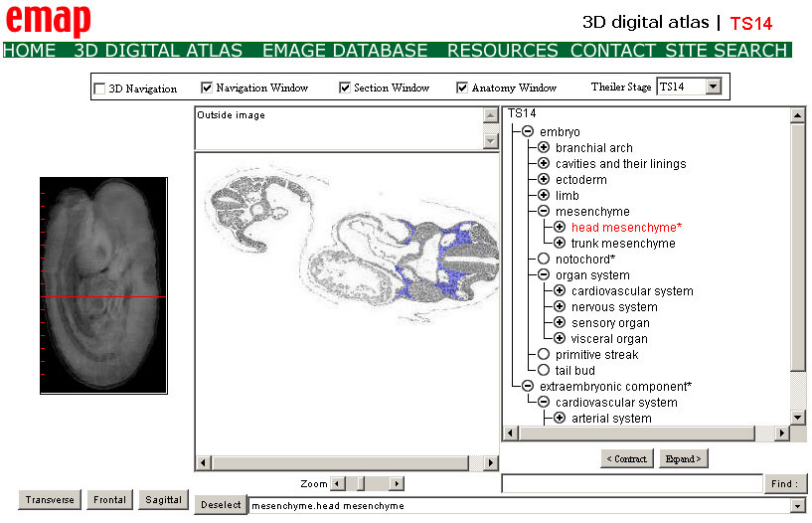
**The EMAP anatomy browser. **The EMAP Anatomy Browser, showing a 3D reconstruction of the mouse embryo at Theiler Stage 14. Components selected on the 2D section (in blue) are highlighted in the corresponding collapsible text index (in red) for that stage. Even with some levels collapsed the inability to obtain an overview of the entire data set can be seen.

The working EMAP browser (see Figure 1) employs a collapsible, indented text index with mappings to corresponding anatomical components in 2D and 3D digital models for the developmental stages of the mouse embryo.

### Extensions required to EMAP browsers

More intuitive methods are required for data analysis, especially where comparisons between multiple data sets, such as lineage across stages, are to be made. A major advantage in visual representation of the data over EMAP's text indices is the availability of an overview in addition to the ability to study ROIs in detail.

The ontology data is structured hierarchically from a root, with unique sub-components together forming complete (super) components [12], using *part-of *relationships. A graphical layout that exploits this structure should aid discovery of relationships within the data [6,8]. Visualisations can make use of physical attributes such as shape, colour and size to encode properties of and relationships between data elements. The following sections discuss advantages in visual data analysis:

#### 1. Representation of complex relationships

As all the developmental stages for a specified anatomy are combined into the complete, abstract organism persistence of components across stages may result in non-unique entries, with different paths to the root.

**Figure 2:**
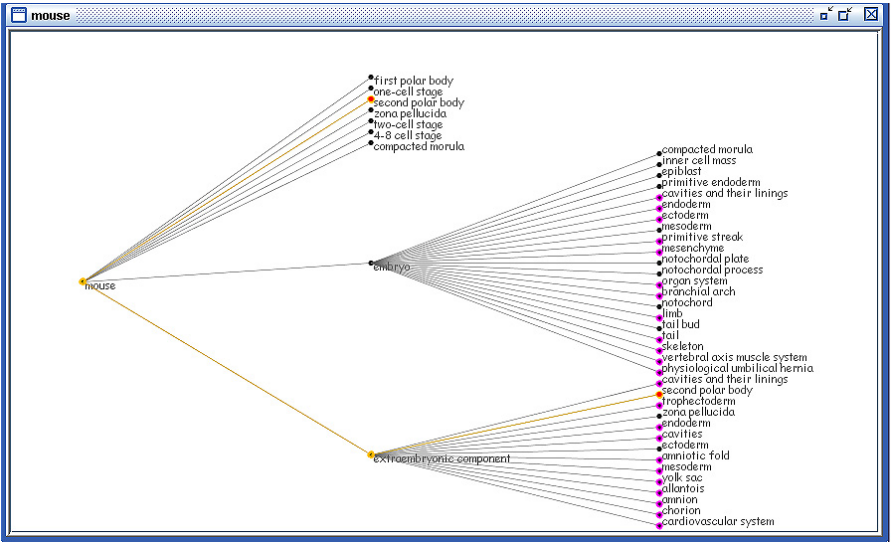
**Multiple parentage for the abstract mouse. **Persistence of components across multiple stages may result in non-unique component names for the abstract organism, but still with unique paths to the root. The *second polar body *is found in different levels in multiple stages. In the abstract mouse it appears twice; the two nodes with identical names are highlighted and the path each traces to the root is drawn.

An illustration from the mouse anatomy ontology would be the component *second polar body*, which exists on the first level below the root for the first three stages of development. However in the 4th stage the *second polar body *forms a part of the *extraembryonic component*. Figure 2 shows the two occurrences of the *second polar body *in the ontology for the abstract organism, on the first and the second levels below the root.

**Figure 3:**
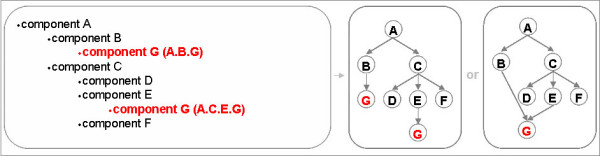
**Employing visualisation for the representation of complex relationships within data. **The collapsible text index contains a component G with multiple parentage; it is necessary to provide two separate text entries to show descent from each parent. The corresponding directed acyclic graph (DAG) could either represent the data with two separate nodes (clones) or display the node G only once, with a link to it from each parent. Advantages in the latter representation include a reduction in the amount of data displayed to users.

The *second polar body *could be regarded as having multiple parentage in the abstract organism, as demonstrated for the component *G *in the index in Figure 3.

#### 2. Grouping

Grouping of anatomical components based on user-specified criteria, to provide different perspectives of the data structure, is a function required for which no graphical support exists in the EMAP browsers. Grouping enables users to focus on relationships within data based on different classification methods for structuring data than those used for constructing the ontologies. A typical example would be the *skin *that covers the entire organism. *Skin *is not represented as a super-component in any one stage; however there may be components in each stage that together make up the *skin *of the organism at that point in development. A group component *skin *could be created that links to all components that comprise the *skin *in a stage, using the default *part-of *relationship again to form a complete whole, while preserving the uniqueness of data components [12] (see Figure 15). Note that this will result in multiple parentage for unique nodes, similar to that occurring in Figures 2 and 3.

#### 3. Lineage

**Figure 4:**
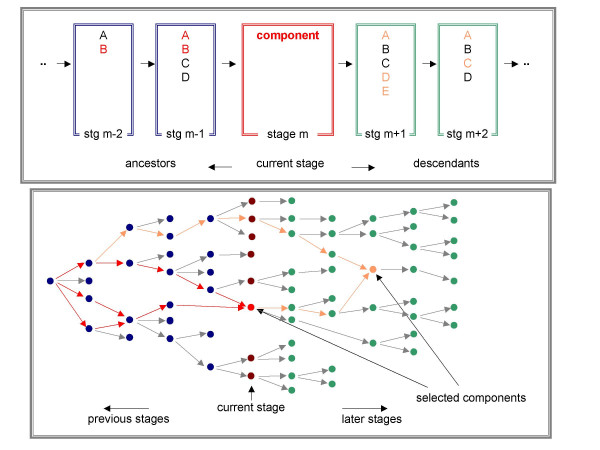
**Using visualisation to trace lineage intuitively. **A more intuitive visual solution to the current textual method (top) for displaying lineage across stages is shown to the right. The lineage paths trace evolution of a component from the root to another component across a number of stages.

Lineage across stages is currently displayed using plain text descriptions of a component's ancestors and descendants in consecutive text boxes arranged along a horizontal plane. The main disadvantage associated with this is the need to scroll through up to 28 text boxes to identify all the Theiler stages (TS) of the mouse embryo, for example, through which a specified component persists (see Figure 4).

#### 4. Visual querying

It is necessary to extend the sub-string searching provided in the EMAP browsers to retrieve related information from external data sources. Lack of integration between databases and the different search and query tools provided for these data sources [1,4] however present problems for information retrieval (IR). Differences in data quality resulting from varied methods for collection and storage, and different data annotation techniques may result in large semantic and syntactic differences in information stored, increasing difficulty in the retrieval and use of data from different sources [13].

**Figure 5:**
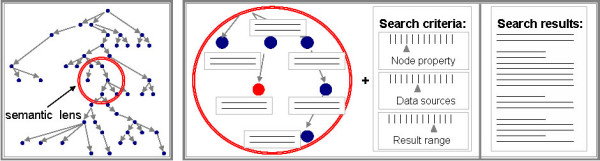
**Visual, dynamic querying. **A semantic lens is used to provide both a physical and semantic zoom. A specified node is then as input for a search and dynamic sliders are used to set search criteria. The provision of search ranges and data sources available aids users in determining information available to perform directed searches. Finally, transparent formatting of search syntax removes the need for users to learn complex query syntax.

Transparent mappings to external data sources that hide their underlying complexity should aid searching and IR. Visual, dynamic querying, illustrated in Figure 5, employing semantic lenses and dynamic query sliders, encourages data exploration by removing the need for users to learn high-level query languages and syntax. Complex queries can be formed intuitively using an interface that provides immediate, visual feedback and allows simplified modification and/or reversal of actions [14], without increasing cognitive load.

Searching currently makes use of free text fields for input, highlighting search hits within the overview. Modification to use a semantic lens could extract information of interest and use this as input for a refined search. The sub-tree containing the component parts of the *heart*, say, could be extracted using a filter that takes *heart *as input and node property *print name *to filter out non-relevant data. Related information from other data sources could then take the results of this search as input to extract gene expression, say, on the components of interest.

#### 5. Simultaneous analysis of multiple anatomy ontologies

Determination of lineage and the comparison of ontologies for different organisms require simultaneous visualisation of multiple data sets. Intuitive comparison between different data sets requires visualisations that highlight mappings between related data elements (see also Figure 14).

### Related work in hierarchical data visualisation

Several applications already exist for the visualisation of large, hierarchically structured data sets. It is important to examine specific applications to determine if users' requirements cannot be satisfied with existing visualisation solutions. Those applications found to most closely approach the requirements of this project are summarised below, detailing features they provide and their limitations for the data analysis required.

#### 1. Protégé

A Java-based knowledge modelling tool, *Protégé *incorporates multiple hierarchical visualisation applications to aid the construction, editing and visualisation of ontologies. These include *OntoViz*, which makes of the *GraphViz *visualisation libraries for graphical representations of hierarchical data. *OWLViz*, specifically developed to visualise OWL ontologies, also makes use of *GraphViz*. A major problem with extending *GraphViz *is that it requires use of the non-standard languages *DOT *and *lefty*.

*TGViz *uses *Touchgraph*, developed in Java, for dynamic layout of nodes in a connected network graph. *TouchGraph *encodes node properties using colour, and provides clustering of like data, as well as geometric and hyperbolic zoom. Functionality for searching and for saving graphs as image files is also provided.

*SHriMP*, providing modular components built using Java Beans, is combined with *Protégé *to form *Jambalaya*, a tool that provides fish-eye views that make use of a continuous zoom for overviews of large data sets. Data abstraction, employing nesting and hiding of data, is followed by extraction of sub-sets to separate windows to allow focus on detail in ROIs. Encoding of data nodes using colour and depth cueing in 3D helps to distinguish more important data. [15]

#### 2. Piccolo

Developed using Java2D and Swing, *Piccolo *provides support for zoom and the use of multiple cameras or viewpoints. *Piccolo *has been customised for visualisation of network and hierarchical data. *GINY*, the Graph Interface Library, extends *Piccolo *to visualise protein and gene interaction and expression in *Cytoscape*. *GINY *uses colour coding of gene expression to aid comparison of data sub-sets. Data reduction is achieved by clustering related data into encapsulating, composite nodes.

*SpaceTree *extends *Piccolo *to produce rooted, node-link hierarchical graphs that combine (physical and semantic) zoom, panning and folding of sub-graphs to provide maximum screen space to ROIs. Main disadvantages of *SpaceTree *include the loss of the overview when analysing ROIs. Also, though the provision of (textual) detail in nodes is useful this reduces screen real estate available for the overview.

*Zoomgraph *extends *Piccolo's *zoomable interface to provide semantic zoom into ROIs. An advantage in Zoomgraph is the ability to define the properties of data nodes and links. [16]

#### 3. Walrus

Using Java3D, *Walrus *employs a hyperbolic projection for the visualisation of directed graphs in 3D space. Although its hyperbolic layout provides an ideal method for displaying the kind of hierarchical data under study *Walrus *is fairly specialised; it uses its own non-standard file format. Further, the structure of graphs once loaded cannot be altered, and only one graph can be loaded at a time. [17]

#### 4. Hypergraph

This Java application provides a hyperbolic layout in 2D that allows interactive repositioning of nodes to provide more magnification to ROIs, with hyperlinks to further detail in external files. [18]

#### 5. VRMLgraph

Developed using Java, *VRMLgraph *draws arbitrary node-edge graphs in 3D. Very little functionality is implemented beyond the drawing of nodes and links connecting them; the main benefit of this application is that it can take advantage of built-in navigation cues and capabilities in VRML for 3D perspective and cameras/viewpoints. [19]

### Motivation for project

The applications and toolkits described above incorporate visualisation techniques for exploration of overviews, and with the exception of *Walrus*, detailed analysis in ROIs. Given the proven capability of hyperbolic layouts for navigation and exploration of large data sets it would be useful to harness the simple but effective implementation of the hyperbolic layout in *Hypergraph*. *Protégé *as it stands provides functionality that would still need adaptation to allow creation of groups as required for the anatomy ontology data. Functionality for folding trees, searching, and highlighting of user paths, and encoding of data properties require further development to satisfy user requirements.

Harnessing existing technology that performs effective analysis of complex data, applied in the tools studied above, will provide some of the functionality required to aid visual data analysis. However it is still necessary to develop novel techniques for analysis of the anatomy ontology data, building on existing methods that have proven useful for visualisation of complex data. Intuitive comparison of multiple data sets and the tracing of lineage through the anatomy ontologies cannot be obtained using the functionality available in 2D tools; occlusion would be too high to allow useful analysis at any level of detail. 3D tools would reduce the problem of occlusion significantly. However 3D visualisations typically distribute data throughout the space available, clustering related data nodes around focal points. Distinguishing individual nodes and the data sets to which they belong is difficult. Drawing physical links between related nodes across data sets may result in crossing of links, reducing the ability to recognise these relationships.

The next section describes the 2D visualisation browser developed that builds on existing techniques to provide intuitive visual data analysis. This is followed by an evaluation carried out to determine if the visualisations provided improve the textual analysis currently performed. Finally a 3D browser develops novel techniques that provide intuitive recognition of relationships across data sets, described in the sub-section **Novel techniques for visual analysis of the ontology data **under **Implementation**.

## Implementation

A prototype has been developed in Java to provide visualisations of the ontology data. A rooted directed acyclic graph (DAG) in 2D provides an overview of each data set, with the ability to study ROIs in detail. Anatomy components are represented using a 2D circle (node), while relationships between nodes are represented by (1D) lines linking nodes, encoding relationships using colour. Functionality implemented is described below, highlighting changes and additions made after the performance of the heuristic evaluation summarised in the next section.

### Description of visualisation prototype developed

#### 1. Layout

Only the top three layers of the DAG are drawn when the visualisation is first generated, in order to provide more screen space for data nodes and minimise occlusion; typical of data with a hierarchical structure, the number of nodes in a level increases as one descends the tree. The top layers provide an abstraction of the data set from which users may continue to extract more detail.

**Figure 6:**
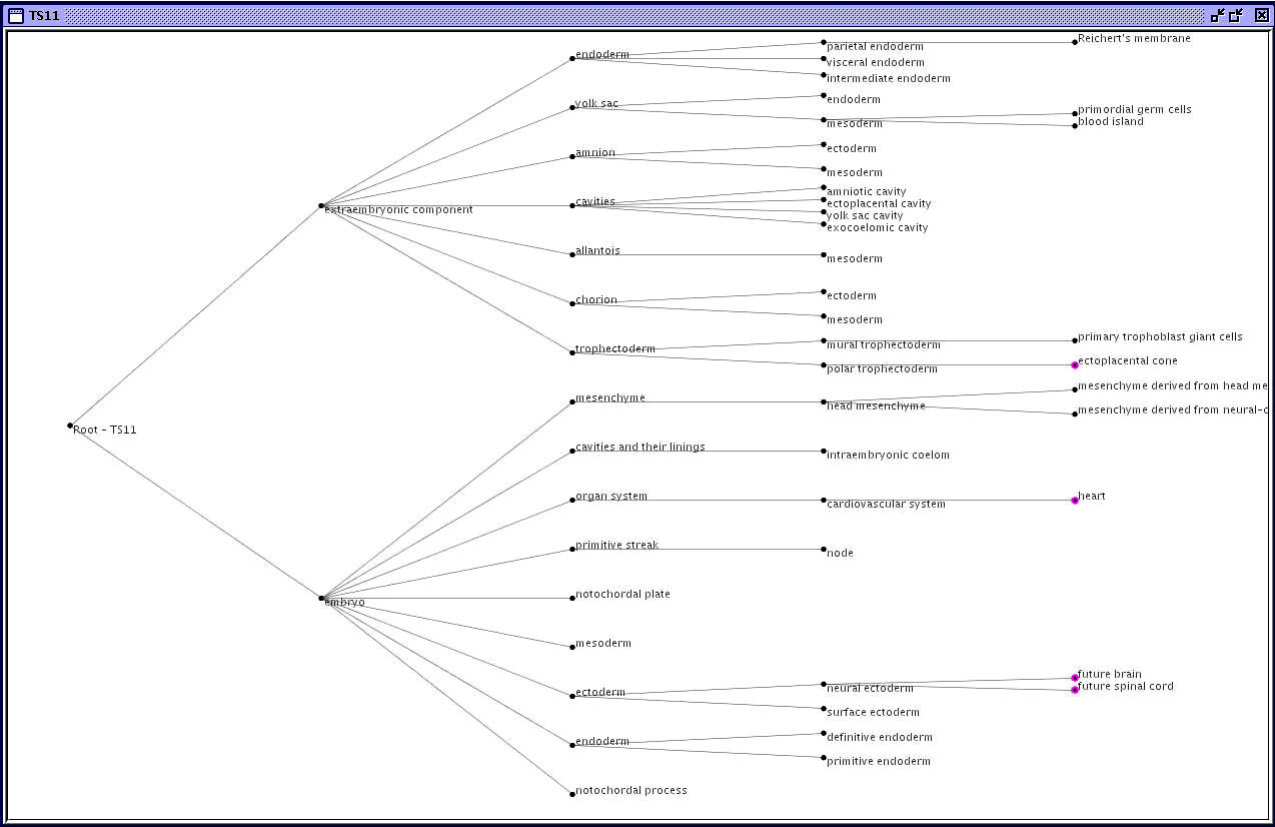
The L-R layout for the first five levels of TS11

**Figure 7:**
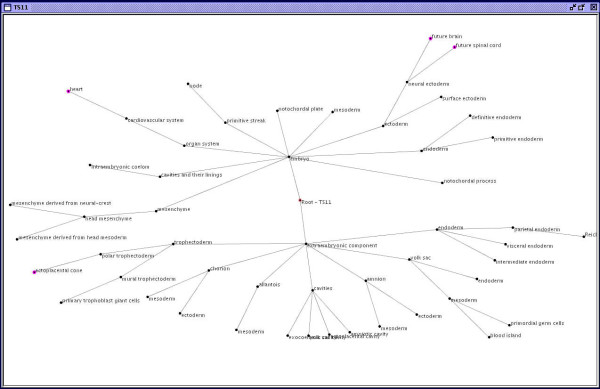
**The equivalent radial layout for TS11 drawn In Figure 6. **There is more uniform distribution of nodes for this layout than for the L-R or T-D. This reduces the problem of high density of nodes in areas of the L-R and T-D layouts while other areas remain largely unoccupied.

**Figure 8:**
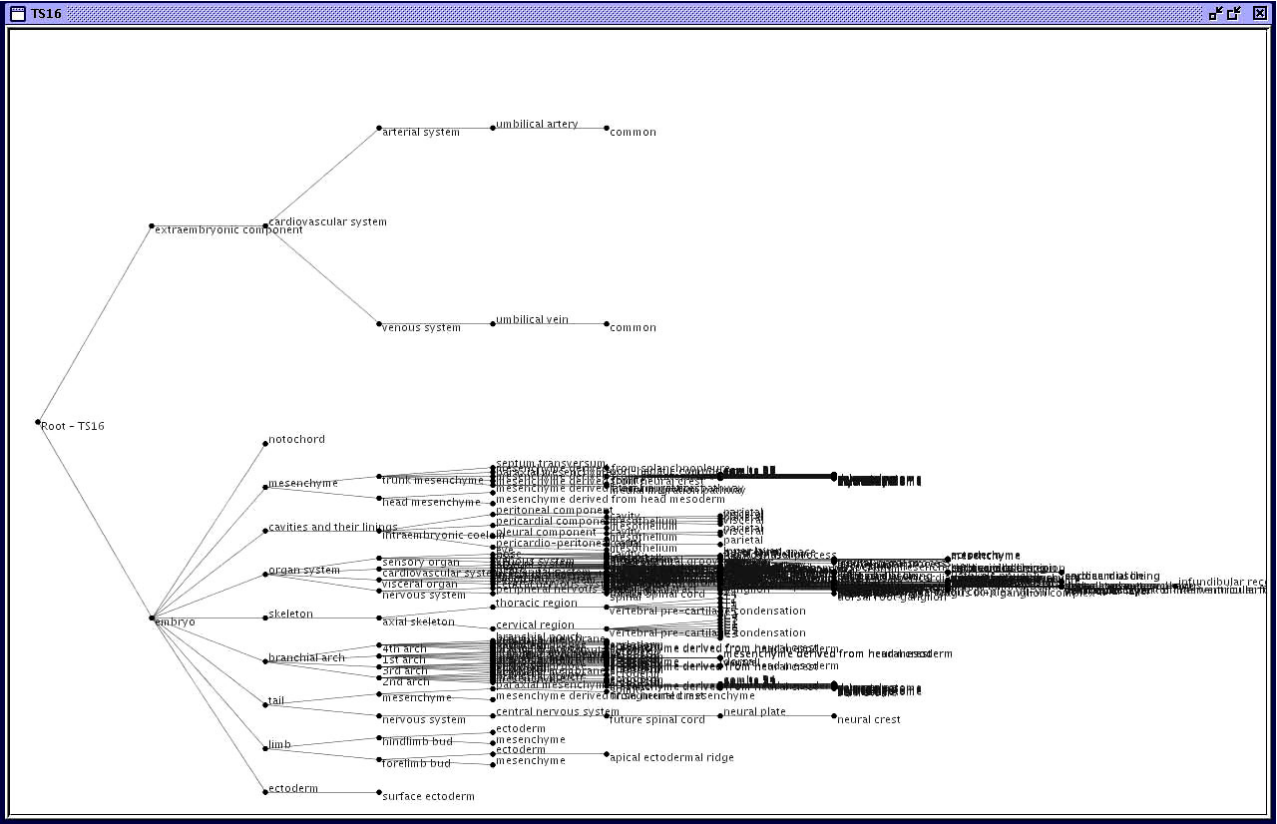
**Unweighted distribution of nodes in L-R layout for TS16. **The bottom half of the graph has a very high level of occlusion; TS16 contains 572 nodes. Without weighting the top half of the graph lies mostly empty, containing only 8 nodes.

**Figure 9:**
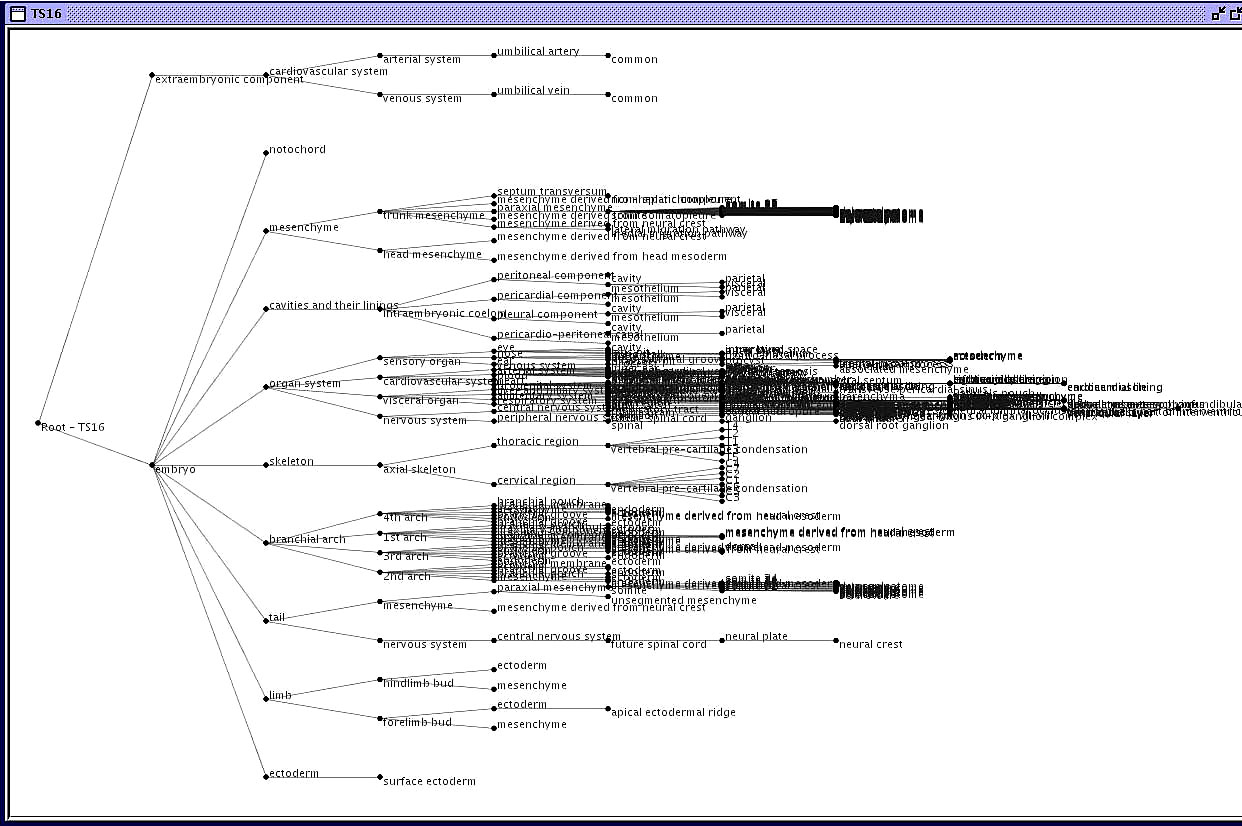
**Weighted distribution of nodes in L-R layout for TS16. **Compared to the unweighted distribution for the same DAG in Figure 8 this layout makes better use of space, aiding analysis in areas in areas of high density by reducing occlusion.

The default layout of the DAG, with the lowest level of occlusion due to node labels, is left-right (L-R), shown in Figure 6. A top-down (T-D) layout is also available. A radial layout, illustrated in Figure 7, was suggested during the heuristic evaluation as an option for more optimal use of screen space. Layout initially provided equal space to all nodes in each level. However as can be seen in Figure 8 this makes poor use of screen space; some areas have high occlusion while others are sparsely populated. The improved layout in Figure 9 weights the layout for the first layer below the root, where the largest bias occurs in node distribution, with weight dependent on number of (immediate) sub-nodes.

#### 2. Textual detail

Labels have a default setting of *component name *for nodes, and (primary) relationship between nodes for links. Labels may be set to any of a node's properties, and all node and link properties may be displayed on request. The option to hide labels may be used to reduce occlusion, in which case holding the mouse over a node or link of interest brings the focus to it and pops up its label.

#### 3. Highlighting & ghosting

Nodes and links of interest may be highlighted for emphasis, while less important data can be suppressed by ghosting it out.

#### 4. Selection of ROIs

A rectangle can be drawn to select nodes in close proximity to each other, making it possible to perform actions on the selection simultaneously.

#### 5. Expansion & collapsing of sub-trees

One solution to occlusion is to collapse sub-trees to hide less relevant data, providing more screen space to visible nodes.

#### 6. Zoom

**Figure 10:**
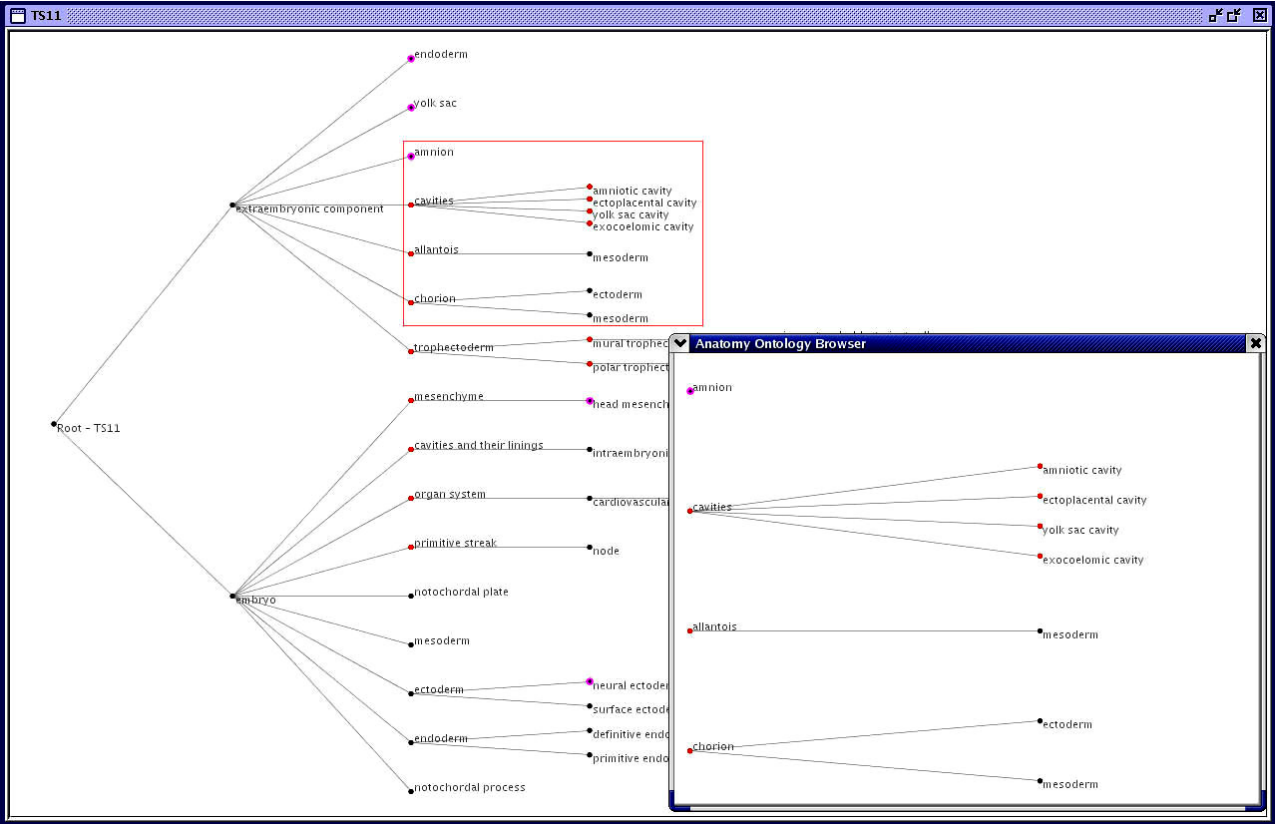
**Zooming into a selection area using a sub-window. **The selection area in the main window is redrawn in a sub-window, providing magnification by drawing the same number of nodes in a larger area.

The prototype has four implementations of zoom: ROIs can be (re)drawn in a separate window, providing magnification by drawing the same number of nodes in a larger area, shown in Figure 10. A sub-tree may also be drawn in a separate window, providing both a semantic and a physical zoom.

**Figure 11:**
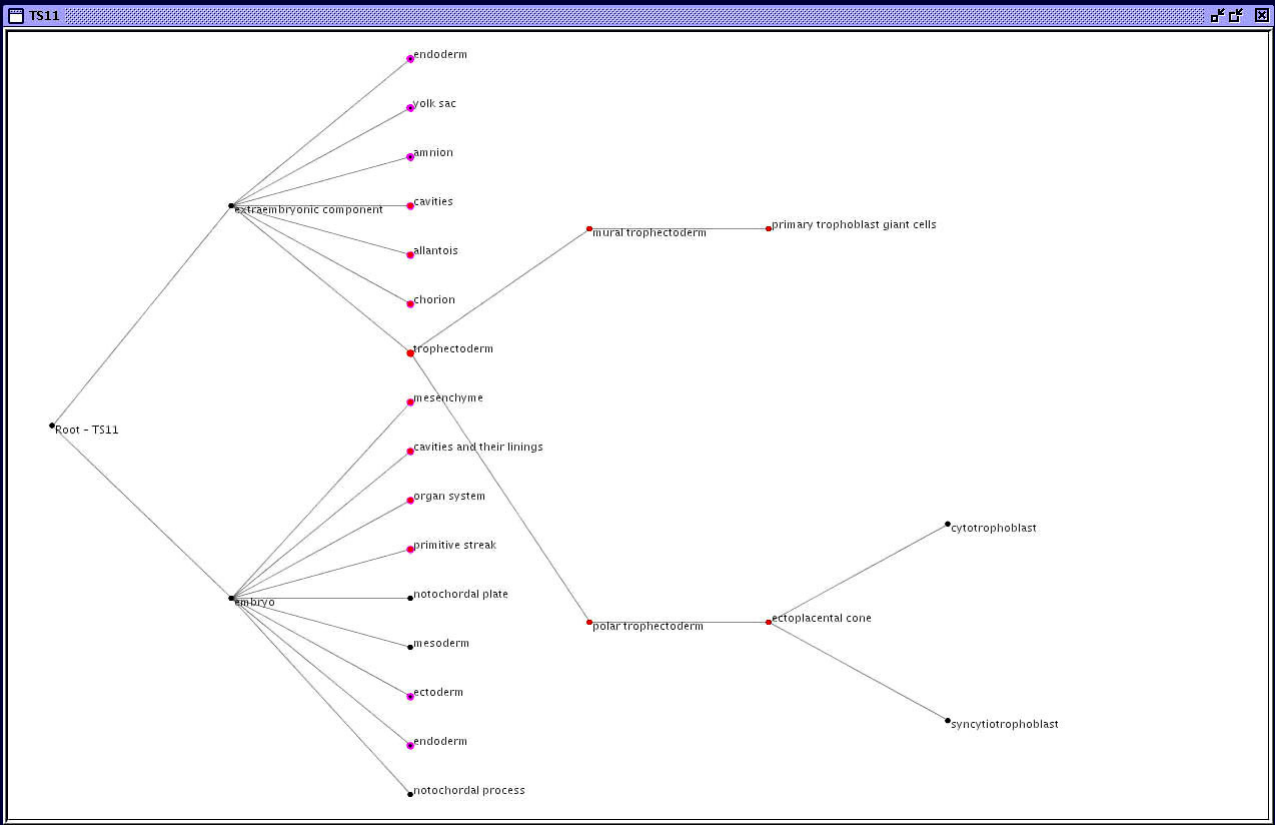
**Semantic and geometric zoom in the main window. **A sub-tree of interest is expanded to take advantage of maximum screen space while suppressing surrounding data of less interest, providing both a semantic and a geometric zoom.

The ability to zoom into an ROI within the context of surrounding information was suggested by users as a useful option. Employing an implementation that makes use of a hybrid between a semantic lens and a continuous hyperbolic zoom, this expands any one sub-tree while collapsing all others beyond that level. The ROI is drawn using maximum screen space, with minimal rearrangement of the graph, as Figure 11 shows. This also removes the extra cognitive load required to map between separate but related visualisations held in multiple windows.

Physical zoom of the entire tree was not implemented initially because this requires scrolling through the DAG to move to ROIs, with an associated loss of context. Users however suggested the reduction in occlusion due to nodes being pushed further apart would make it easier to explore detail in ROIs, compensating for the loss of the overview. This option has now been implemented.

#### 7. Searching/querying

A custom dialog allows users to perform sub-string searches on any of a node's properties. Textual results showing component ID and print name for nodes that satisfy a query are supplemented with highlighting of hits in the graph.

#### 8. Grouping of nodes

Functionality is provided for the creation of *group nodes*, linked to nodes already existing in the DAG, to group related nodes (see **Grouping **under the section **Extensions required to EMAP browsers **and Figure 15).

#### 9. Tracing lineage

Graphical layout of the data aids determination of lineage by allowing users to trace the ancestors or descendants of a node using a data overview.

#### 10. Resetting to default state

The option to remove all formatting applied to nodes returns to the default rendering of data elements.

### Heuristic evaluation of the 2D anatomy browser

A heuristic evaluation was carried out with a small group of target users comprising biologists and computer scientists working at the MRC on EMAP. This involved a demonstration of the functionality available for interaction with and manipulation of the visualisations generated. Users commented on usefulness (or not) of aspects of the system, and made suggestions for improvements, additions and changes to the system. This led to the redesign and re-implementation of the prototype, in preparation for a structured user evaluation.

A major issue encountered in the visualisation of complex data is occlusion (see Figure 8), an acute problem in the visualisation solution developed, especially due to data labels. The evaluation highlighted further issues with occlusion in the visualisations, and provided suggestions for reduction of this problem, illustrated in Figures 6, 7, 9, 10 and 11.

**Figure 12:**
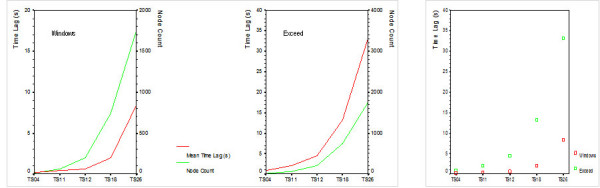
**System response time with load for the 2D browser. **Plots showing the exponential increase in system response time with data load for the 2D browser.

A second major problem identified is the exponential increase in system response time with data load, with a significant negative impact on interaction, illustrated in the graphs in Figure 12. This problem is even more acute for remote execution in X-windows, probably due to enhancements for Swing in Windows which have the reverse effect in X-windows [20].

### Novel techniques for visual analysis of the ontology data

**Figure 13:**
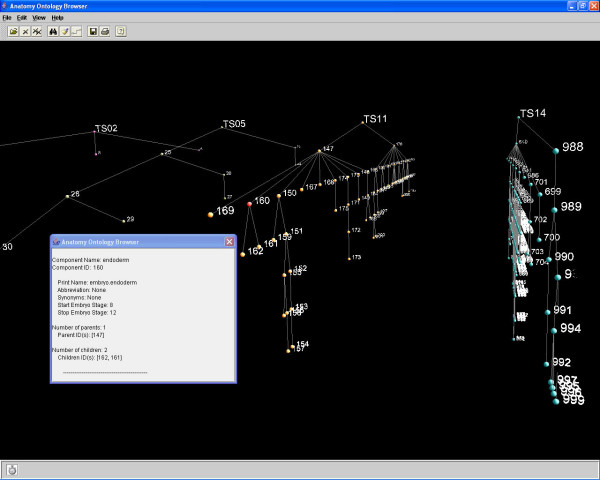
**Extension to 3D to allow simultaneous visualisation of multiple datasets. **Simultaneous display of four stages in a 3D window; DAGs are laid out in parallel planes equidistant from each other, with all root nodes lying in the same plane and the DAG growing downwards. The snapshot zooms into an ROI and shows textual detail brought up for a selected node (highlighted in red).

The main advantages in 3D visualisation of large data sets are the larger amount of space available for data display, due to the added dimension of depth, and the higher degree of freedom for exploration and navigation. Natural perspective in 3D also provides increased magnification as one approaches the user's viewpoint. Using 3D makes possible the simultaneous display of multiple data sets (see Figure 13), with far less occlusion than would occur in 2D.

This project requires the analysis of multiple data sets, to identify relationships that span multiple stages in one organism or map relationships between different organisms. Beyond a threshold of about 200 nodes, however, significant occlusion occurs in 2D.

The 2D browser makes use of existing techniques for visual analysis of individual data sets, with modifications and extensions as required to provide optimal analysis. To improve data analysis in areas of high occlusion different kinds of zoom have been implemented, including the development of a hybrid lens (see **Zoom **under the section **Description of visualisation prototype developed**). A uniform zoom expands a sub-tree of interest to make maximum use of space in the main window; without relocation surrounding nodes would be obscured, as occurs for magic lenses. A hyperbolic lens could be used to move surrounding data to the periphery of the window; however constantly changing hyperbolic layouts have the disadvantage of destroying the mental models users form of data structure. The hybrid *lens *developed folds nodes on the same level as the node whose sub-tree gains the focus. Surrounding context is still maintained to a large degree, while users concentrate on analysis of the ROI. However this and other methods for reducing occlusion in the 2D browser provide only a partial solution, and only for individual data sets.

Increasing to three the number of dimensions used for visualisation provides more space in which to hold data notes, further reducing occlusion of data. 3D however comes with its disadvantages, detailed in the following section, with the most significant being disorientation when navigating through 3D space. Evidence however exists for the increased usability of between 2.n (n > 0) and 3 dimensional [8] visualisation of data; users are able to move out of the area holding the data and *fly over *or below ROIs. This reduces the feeling of immersion into the data and the disorientation this causes. Overviews of data sets are obtained that improve users' mental models of data structure, and users are able to move back into the data as required to analyse ROIs in detail.

**Figure 14:**
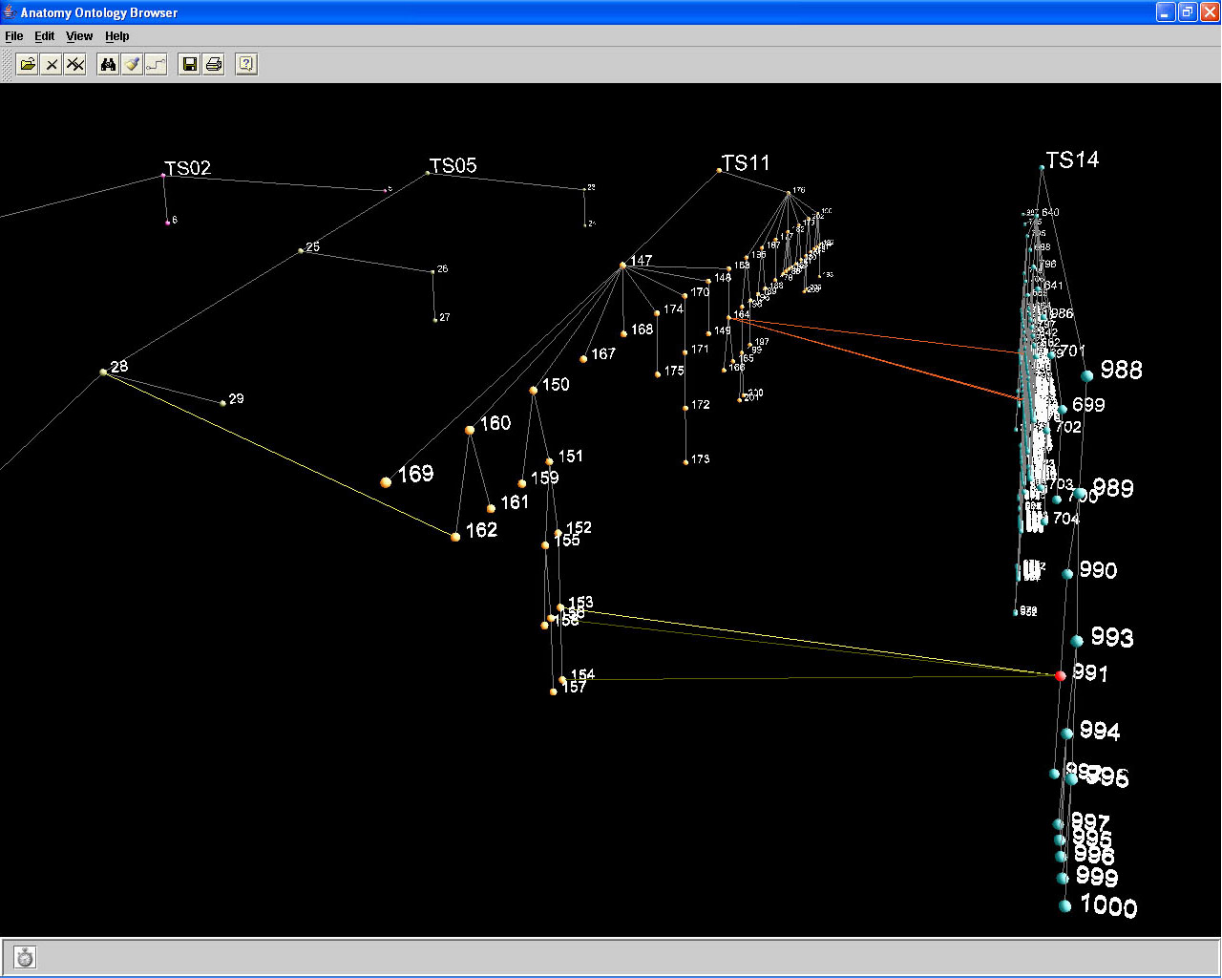
**Relationships spanning data sets in the 3D browser. **Colour-coded links are drawn between three DAGs to show relationships across stages, functionality that can also be used for tracing lineage.

In order to take advantage of the space provided in the extra dimension without losing the benefit of simpler analysis in 2D a novel system has been developed that continues to draw individual data sets in 2D, layered uniformly in parallel along the horizontal axis (see Figure 13). The physical space between the layered DAGs serves as the information space used to hold relationships between node pairs belonging to different ontologies. Links are drawn across the space between related data sets, using colour codes to represent the different relationships that exist. Users are able to transfer learning in the use of the 2D browser to visualisation in 3D, continuing to analyse individual data sets in relative isolation, each lying in its own plane. Because relationships spanning data sets lie in separate planes and are drawn parallel to those holding the DAGs they stand out and are easily recognised, especially when viewed from a point above or below the data area, shown in Figure 14.

**Figure 15:**
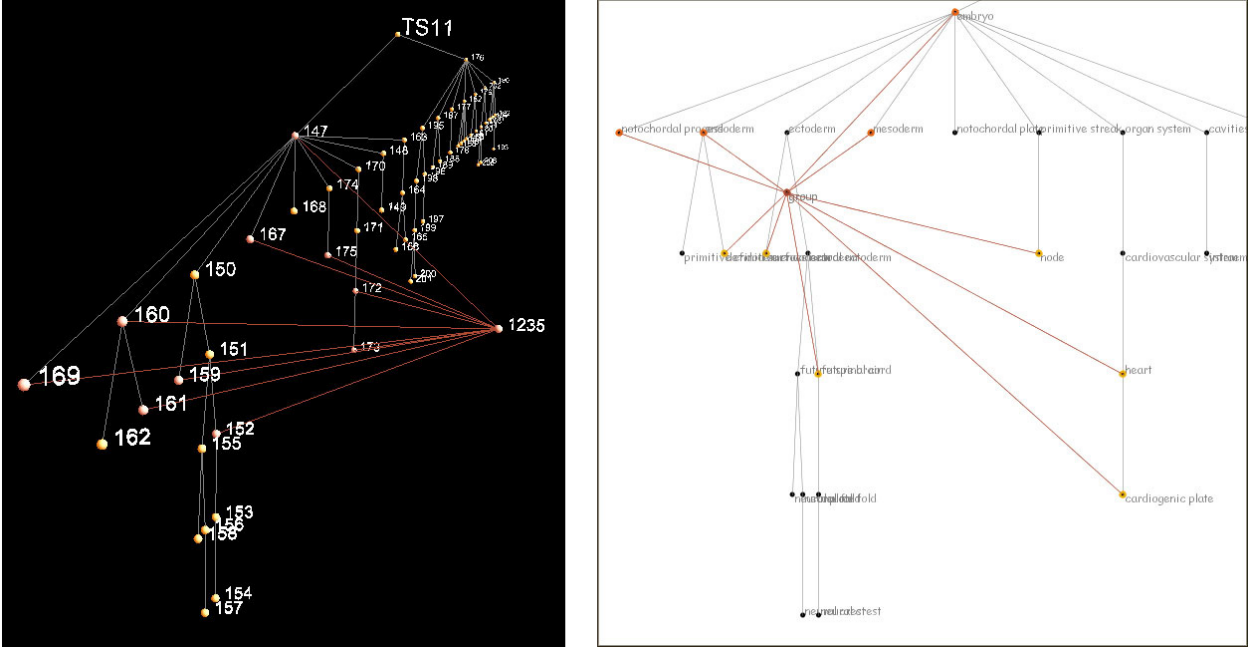
**Comparison of visualisation of grouping in 3D to 2D. **Removing the group node to a plane parallel to that in which the DAG lies (in 3D) not only makes it easier to identify the group created, but also prevents the crossing of links that occurs for grouping in the 2D browser.

Figure 15 illustrates the solution implemented for grouping of nodes in the 3D anatomy browser. This shows the benefits gained by relocating the group created to a plane parallel to that in which the DAG lies, removing the crossing of links that occurs in 2D. Placing group nodes in a separate plane has the added advantage of removing the increase in occlusion that occurs in 2D due to the addition of further nodes and links in the plane holding the DAG. The **Structured evaluation of the visualisation prototypes **detailed further in this paper confirmed that grouping of nodes in 3D was found to be more intuitive than for the 2D browser.

### Issues encountered in the 3D visualisations

The main difficulty encountered interacting with the 3D browser is maintaining control over the data structure during navigation. Built-in functionality for navigation in Java3D employs the keyboard and/or the mouse, allowing translation, rotation and zoom. The lack of a history function means that it is difficult to return to specific points in the data structure; the only option available to users for recovery when the data structure is moved out of the viewing area and the bounding sphere within which behaviours are active, or when users become lost in the data, is to return to the centre of the universe (and default camera viewpoint).

Occlusion of more distant data in 3D, disorientation of users during navigation, and the complexity associated with the creation and support of 3D visualisations in terms of software and hardware required mean that 3D may not necessarily provide a better option than 2D. Employing 2D for the visualisation of individual stages or for abstracted views of individual, complete anatomy ontologies, with an extension to 3D as multiple stages and/or ontologies are compared may provide an optimal solution. Functionality for switching between the 2D and 3D views should help to resolve the disorientation that occurs in 3D by allowing users to focus on only data of interest in 2D without the distraction of extraneous information. Continuing to place individual data sets in 2D planes reduces disorientation by giving users more control over the level to which they become immersed in the data; users are able to move above the area holding the data and observe its structure from above or below. This provides an overview that highlights all relationships occurring across data sets in addition to displaying each data set lying in its own 2D plane.

To ease integration of the prototypes with the EMAP Anatomy Browser it is important that their graphical user interfaces (GUIs) do not deviate significantly from that of the EMAP browser. This should reduce users' learning curves, increasing willingness to use the prototypes. For this reason it may be useful to continue especially GUI development in Java. This also eliminates the problem of cross-platform compatibility and enables development for use on the Internet. Java3D, using OpenGL for low level rendering acceleration, may seem the obvious choice for the extension of the 2D browser. However limited support for development using Java3D may result in restrictions in functionality and that may have little future support. Further, Java's cross-platform compatibility comes with a cost in performance due to the overhead incurred in the interpretation of Java byte code into native machine code, manifested in the large increase in system response time observed as data load increases.

Another option would be to develop the 3D browser using OpenGL (with C/C++), to take advantage of OpenGL's advanced 3D modelling capability, hardware acceleration and larger user base and support. GL4Java bindings from OpenGL to Java could then be used to provide an interface with the benefits of Java.

### Structured evaluation of the visualisation prototypes

In order to establish if user requirements have been fully and correctly captured and implemented it is necessary to evaluate the visualisation prototypes with target users. This provides measures of user satisfaction and determines the effectiveness and efficiency of the systems developed. If the new system developed is to be used it will have to provide advantages over tools in current use, improving productivity without increasing users' work load [7,20,21]. A structured evaluation should identify usability issues and highlight features that improve data analysis. Requirements for changes to, additions and extensions to functionality also need to be identified. Sources of error and poor system response should be minimised; effective system feedback and good error management contribute to user satisfaction [5].

To guide the evaluation two main hypotheses were tested:

H_0_: Visual analysis of especially large, complex data sets provides advantages over textual analysis.

H_1_: Visualisation in 3D provides advantages for analysis over 2D that justify the larger amount of support required.

### Preparation for structured evaluation

#### 1 Task scenarios

To perform optimal data analysis users must possess the information required to achieve their goals: domain knowledge and how functionality in systems maps to this. To be effective a user evaluation should simulate the working processes of typical users in their normal working environments. A set of task scenarios were developed, detailing successful completion criteria and maximum goal completion times. These benchmarks could then be used to compare expected/ideal user behaviour to actual.

A heuristic evaluation, involving a walk through the scenarios developed, was carried out to ascertain that the scenarios capture typical user tasks in a normal work environment and allow users to explore functionality offered. The walk-through also highlighted further development required to increase intuitiveness of the prototypes, summarised in Table 1 below, and carried out prior to the structured evaluation.

#### 2 Custom questionnaires

Custom questionnaires based on the QUIS, the Questionnaire for User Interface Satisfaction [5], were prepared to elicit subjective measures of user satisfaction and usability of the prototypes: a pre-evaluation questionnaire to collect demographic information and a post-evaluation questionnaire to collect information on usability of the system. The (custom) questionnaires were reviewed by an HCI (Human Computer Interaction) expert and revised where necessary.

#### 3 Pilot test & review of evaluation procedure

A pilot test was then carried out with an (independent) HCI expert with a specialisation in visualisation. This examined the design of the evaluation procedure and checked that usability requirements had been integrated into it.

## Results and discussion

### Implementation of structured evaluation procedure

#### 1 User backgrounds

Two main target user groups of the prototypes have been identified: the primary target – biologists, and the secondary target – computer scientists working in bioinformatics.

Ten users in total performed the evaluation, six from the MRC and the remaining four, research students at the School of Maths and Computer Sciences (MACS) at Heriot-Watt University. With the exception of one user, an HCI expert from MACS, all users had some experience in bioinformatics, and most had at least tried out the EMAP browsers and/or had some association with XSPAN (The Cross Species Anatomy Network).

#### 2 Methodology

A flow diagram was used to record users' paths to complete each task, noting additionally, users' reactions and comments, errors made, requests for help and responses given. Software logging captured interaction with the menus and toolbar (transparently), and a built-in timer was used to record task completion times.

Verbal help was provided by the developer to supplement the help files which had not been completed prior to the structured evaluation.

### Analysis of evaluation data

Qualitative feedback was obtained from the custom and the SUS (System Usability Scale) questionnaires administered, and from observation of users recorded during each evaluation. Because the test user population is only ten statistical analyses may not provide very reliable indications of satisfaction or otherwise with the prototypes. Therefore the only statistical tests performed are those looking at means (within a 95% confidence interval (CI)) and extreme values of rankings. Mean user satisfaction rankings for the post evaluation questionnaire, SUS scores, and task completion times are shown below:

#### 1 SUS scores

**Figure 16:**
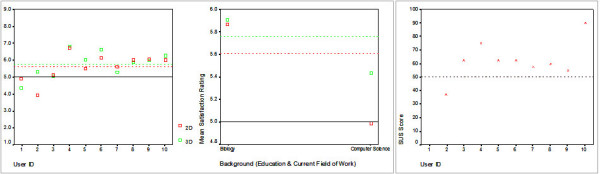
**User satisfaction ratings for the structured evaluation of the visualisation prototypes. **The two charts on the left show the mean overall satisfaction ratings for each of the 2D and 3D browsers. The first chart shows means for each user and the second, means by target user group. The colour coded reference lines for each chart show the mean overall satisfaction rating for each browser, while the black reference line shows the midpoint for the Likert scale (ranked 1–9) for the questions posed. The third chart plots SUS scores for each user (note there is no score for user 1).

The SUS scores obtained, as shown in Figure 16, indicate eight out of nine users with a score above the mid mark (50). The highest score obtained was 90 (out of 100) and the lowest was 37.5. The mean SUS score is 62.5.

#### 2 Satisfaction ratings

Overall means for satisfaction ratings for individual users, shown in Figure 16, have eight out of ten users with rankings above the central mark 5, with five of those values lying above the mean for the entire sample. One user ranked the 3D browser above the central mark and the 2D below, and the last ranked both below the central mark. Mean rankings for the 3D browser were higher than for the 2D, both over the whole user population and by user group (see Figure 16). Rankings by target user group saw biologists, the main target, rating usability higher than computer scientists.

Further analysis of (mean) rankings for each item show quietness of system, consistency of terms used and good relation to users' normal work, eased ability to determine lineage, low time to achieve proficiency using the systems, eased data analysis, and the visualisations providing advantages over the text indices fell in the top ten for both the 2D and 3D browsers. Hiding of sub-trees to reduce occlusion and reliability of the system were other items ranked in the top ten for the 2D browser. Consistency of messages on screen, and the options for zoom also fell in the top ten rankings for the 3D browser.

Large variations in system speed had the worst ranking for both browsers, followed by average time to perform tasks. Occlusion of data, level of support for error recovery, ease of navigation through data, and flexibility of the system all fell within the lowest ranked items for both browsers. Additionally, for the 2D browser, the system being satisfying, ease of reading text on screen, ghosting of nodes and occlusion of data specifically for the T-D layout fell in the bottom ten rankings. Ability to identify errors and their sources, difficulty of the system and the needs of experienced and inexperienced users being taken into account all fell within the bottom ten rankings for the 3D browser.

#### 3 Task completion times

**Figure 17:**
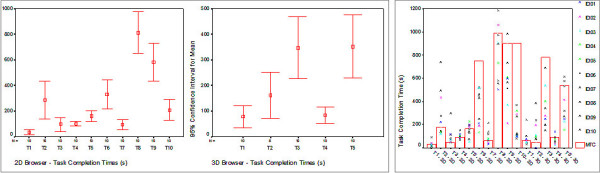
**Task completion times for the structured evaluation. **The two charts on the left show mean task completion times within a 95% CI, while the third plots task completion times for each task for each user, compared to the expected maximum time (MTC) to complete each task.

Task completion times are summarised in Figure 17.

It is observed that times for T2-2D (task 2, 2D browser), T3-2D, T7-2D, T1-3D and T2-3D are very high compared to expected maximum completion times. Analysis of the qualitative feedback from the flow diagrams for each user provides clues that explain these results, highlighting areas where users had difficulty identifying or understanding functionality required to complete tasks. Restrictions for data input, poor wording or ambiguity in function labels and poor presentation of additional textual information to users contributed to most of the problems encountered. Learning was exhibited by users performing repeated tasks in 3D in shorter times than expected. A significant example is for grouping of nodes; users also found grouping in 3D to be more intuitive than for the 2D browser (see section on **Novel techniques for visual analysis of the ontology data**).

### Changes made to prototypes based on structured evaluation

A number of changes and additions have been made to the prototypes, based on the evaluation results. More detailed information is provided on node and link properties, and functionality is available for insertion of user comments. The DAG now automatically displays more levels if required to uncover hidden information requested by users. Further options for search include searching on hidden nodes and within a sub-tree only. Specific search hits in the DAG may also be isolated and highlighted from within the search dialog. Ghosting now fades out links to and from ghosted nodes and hides their labels.

The functions for creating groups have been moved to the Edit from the View menu, to map to users' semantic interpretation of the function. Options for selection of nodes now include retrieval from the graph by clicking, choosing from a drop-down list or by entering component IDs in a free text field. Functionality for editing and removing groups has also been implemented, and users may extract a group of interest for analysis in isolation in a 2D (sub) window.

Input dialogs when first opened now prompt users with the current value of the variable of interest. Where practicable free text fields have been replaced with sliders to prevent errors by allowing input only within legal ranges. An editable legend detailing encoding properties for nodes and links has been provided.

Full implementation of functionality in sub-windows allows tighter coupling between the main and sub-windows. Functionality for switching between the 2D and 3D browsers without exiting either application has been implemented. The ability to save user sessions (to text or image files) aids incremental analysis. Finally, the help files now also provide implementation detail and clarification of ambiguous terminology.

## Conclusion

The EMAP browsers in current use employ textual indices for the analysis of anatomy ontology data for the mouse embryo, with mappings to 2D slices in 3D regions of models of the embryos at each stage of development. The lack of an overview for these large data sets increases users' cognitive load during data analysis; more intuitive methods for analysis are required to aid research.

Visualisation of large, complex data sets exploits human perceptual abilities to increase cognition and ease data analysis, by providing spatial representations that map to users' mental models of data structure. This paper explores the use of hierarchical graphs for the visualisation and analysis of the complex, hierarchically structured, multi-dimensional ontology data under study, assessing different visualisation techniques available for analysis of this data and their merits and limitations. Prototypes are being developed for 2D and 3D visual analysis of the ontologies, to provide alternative and novel solutions where methods and techniques available are unable to meet users' needs. Visualisations generated provide overviews of each data set with functionality for further interactive analysis of ROIs.

Evaluation of the prototypes being developed aimed to test two main hypotheses: whether visualisation provides advantages over textual representation for data analysis, and whether 3D visualisation provides enough advantages over 2D to justify the larger amount of development and support required for it.

Analysis of the data collected during the heuristic and structured evaluations showed users found the visual representations of the data to provide significant advantages over text for determining data structure, and more intuitive methods for search and query. However increasing levels of occlusion with data set size render visualisations of the larger data sets difficult to use. Functionality for data reduction and the ability to analyse data of interest in isolation offer improved solutions for analysis. Further novel solutions are required to resolve more fully the problem of occlusion.

Evaluation results showed the 3D browser slightly more intuitive for use than the 2D. The larger amount of space available in 3D makes it possible to display more data with a lower amount of occlusion than occurs in 2D. Functions for searching, grouping of data and locating information using the 3D browser were also found to be more intuitive. The large variations in system response time encountered in 2D, a major source of user dissatisfaction, did not pose as significant a problem in 3D.

## Further work

Further functionality for the 3D browser will therefore be implemented, looking especially at providing solutions for the most significant problems encountered in 2D.

Further development on the prototypes will build visual structures that provide effective, interactive encoding and display of multiple relationships between and within different data sets.

### General applicability of software developed

The visualisation applications have been developed to work with data from EMAP, stored in XML or HTML files. Storage in self-describing XML eases analysis of the data structure; the information obtained is used to generate the visual representations that provide intuitive analysis of the anatomy ontologies under study.

The next stage of the project is looking at reading data directly from the EMAGE (Edinburgh Mouse Atlas of Gene Expression) database. The browsers are built so that a layer exists between the data read in and the generation of the visualisations. Therefore reading in data from different sources only requires writing additional parsers for the new data sources, and feeding the parsed data into the visualisation system.

The visualisation browsers however expect specific data properties; the systems also require updating to read in and process additional or different data properties from those used to describe the structure of the mouse embryos, to allow customisation for differently structured ontologies. Visualisations for other data sources containing ontology data may then be generated after writing custom parsers for the data they store.

## Availability and requirements

**Project names: **EMAP and XSPAN

**Project home page: **

**Operating system(s): **Platform independent

**Programming language: **Java

**Other requirements: **Java3D with OpenGL

**License: **None

**Any restrictions to use by non-academics: **None

## Authors' contributions

ASD did the background research, built the prototypes and carried out the evaluations, and wrote the paper. AB was one of the reviewers in the heuristic evaluation, provided suggestions in building the prototypes, reviewed the draft of the article and approved submission.

## Figures and Tables

**Table 1:** Proposals for changes to browsers prior to structured evaluation; changes implemented before evaluation are flagged.

**Original implementation**	**Suggestion(s) for improvement**	**Implemented**
**Occlusion due to node labels: **occurs even where occlusion of nodes is very low to none, especially for TD layout of the DAG	1. Hiding of labels (already implemented) 2. Interactive repositioning of nodes/labels 3. Drawing labels at a (user-defined) angle to horizontal plane	√ X X

**Default labelling of nodes: **set to component name	Change to print name (full path to root) to aid differentiation between nodes with identical component names, e.g., TS12 has four nodes with component name *mesenchyme*. (Note that this solution increases the problem of occlusion due to node labels)	√ (implemented in some cases)

**Component detail: **only component IDs provided in some cases	Provision of print names in addition to component IDs, as otherwise required to look up names to identify nodes	√ (partially implemented)

**Search:**	Ability to highlight a specific search hit from within search dialog	√

**Creation of groups: **group nodes could only be selected from list held in dialog	Ability to click to select group nodes using the DAG Ability to enter component IDs directly	√ X

**History/Undo function: **not available	Undo function encourages exploration, especially for 3D where navigation sometimes produces unexpected, undesirable results	X

**Storage of user sessions: **not available	Storage of user sessions – provides history function	√
